# Comparison of complication rates in transgender top surgery (female to male) between conventional bandages and negative pressure wound therapy – A retrospective analysis

**DOI:** 10.1016/j.sopen.2025.03.003

**Published:** 2025-03-14

**Authors:** Carmen Leser, Georg Dorffner, Fiona Kabashi, Christine Deutschmann, Daniel König, Zaza Kashibadze, Selina Ebner, Daphne Gschwantler-Kaulich

**Affiliations:** a Department of Obstetrics and Gynecology, Cancer Comprehensive Center, Medical University of Vienna, Vienna, Austria; b Section for Artificial Intelligence and Decision Support, Medical University of Vienna, Vienna, Austria; c Clinical Division of Social Psychiatry, Department of Psychiatry and Psychotherapy, Medical University of Vienna, Vienna, Austria

**Keywords:** Transgender top surgery, Breast operations, Negative pressure wound therapy, Complication rates, body contouring

## Abstract

Fifty to 70 % of transgender patients undergo gender-affirming top surgery. In other types of surgeries, the use of negative pressure wound therapy (NPWT) was described as a major point in reducing complications, and we, therefore, examined possible similar effects when using it in gender-affirming top surgery.

We investigated differences in the complication rates after body contouring surgery with or without the use of NPWT and included 58 female-to-male transgender patients who have been operated on at the Medical University of Vienna between 2017 and 2020 in this retrospective analysis.

Without NPWT, significantly more patients suffered from wound dehiscence (*p* = 0.026) and slightly more patients had to undergo postoperative percutaneous drainage due to seroma (*p* = 0.129). However, patients had significantly less revision surgery because of severe bleeding with the conventional dressing (*p* = 0.005). The surgical method was another factor influencing the occurrence of wound dehiscence, especially regarding the incision type and the resected volume. Large breasts and the necessity for using a typical mastectomy incision were underlying factors for dehiscence.

There are fewer complications when using NPWT, especially regarding wound dehiscence in top surgery; however, postsurgery monitoring is required for severe bleeding afterward.

## Introduction

50–70 % of transgender patients decide to undergo gender affirming breast surgery [1].

Before surgery, the majority of the patients are administered hormones for gender affirmation. Due to increased complication rates, many surgeons provide cessation of gender-affirming hormone therapy prior to chest surgery. Reasons for this cessation include fear of severe bleeding, hematoma, and thrombotic events [2]. No difference was observed in intra- and postoperative complication rates continuing or discontinuing hormone therapy [3] (Table 1a, Table 1b, Table 2, Table 3).

**Table 1a:** Patient characteristics.

Characteristic	Conventional dressing	Negative pressure wound therapy	p value
N	35	23	
Age	26.7 (8.5)	24.1 (5.4)	0.453
BMI	24.8 (3.5)	23.0 (4.9)	0.026
Smoking status			
Nonsmoker	22 (62.9 %)	20 (87.0 %)	0.148
Ex-smoker	4 (11.4 %)	1 (4.3 %)	
Smoker	9 (25.7 %)	2 (8.7 %)	
Comorbidities	12 (34.3 %)	4 (17.4 %)	0.159
Surgical technique			
periareloar scar	28 (80.0 %)	15 (65.2 %)	0.208
DIFNG scar	7 (20.0 %)	8 (34.8 %)	
Length of hospital stay (days)	6.6 (1.1)	6.8 (2.2)	0.726
Length of hormonal treatment (months)	19.7 (9.3)	16.9 (6.6)	0.301

**Table 1b:** Patient characteristics.

Characteristic	Periareolar	DIFNG	p value
N	43	15	
Age	24.2 (6.0)	29.7 (9.7)	0.030
BMI	23.1 (3.4)	26.9 (4.9)	0.011
Smoking status			
Nonsmoker	31 (72.1 %)	11 (73.3 %)	0.616
Ex-smoker	3 (7.0 %)	2 (13.3 %)	
Smoker	9 (20.9 %)	2 (13.3 %)	
Comorbidities			
Length of hospital stay (days)	6.4 (1.2)	7.3 (2.4)	0.466
Length of hormonal treatment (months)	18.1 (8.3)	20.2 (9.0)	0.222

BMI, body mass index; DIFNG, double incision breast reduction.

**Table 2:** Complications.

Characteristic	Conventional dressing	Negative pressure wound therapy	p value
N	35	23	
Revision/bleeding	1 (2.9 %)	7 (30.4 %)	0.005
Puncture	7 (20.0 %)	1 (4.3 %)	0.129
Dehiscences	12 (34.3 %)	2 (8.7 %)	0.026

**Table 3:** Surgery techniques.

Characteristic	periareolar	DIFNG	p value
N	43	15	
Revision/bleeding	7 (16.3 %)	1 (6.7 %)	0.666
Puncture	6 (14.0 %)	2 (13.3 %)	1.000
Dehiscences	13 (30.2 %)	1 (6.7 %)	0.087

To plan a top surgery, a documented gender-affirming dysphoria is required. Surgical techniques depend on the volume, shape and ptosis of the breast. For larger breasts, a double incision mastectomy with free nipple grafting (DIFNG) is common, while a mastectomy with periareolar skin reduction should be planned in smaller breasts. Each technique has its advantages and disadvantages. While an DIFNG has a longer scar, the patients mostly only require a single operation. With the periareolar technique, the patients benefit from a smaller scar, but they often have a kind of revision surgery, for example, scar correction or additional skin reductions [1].

Negative pressure wound therapy (NPWT) is widely used for many types of wounds [5]. Vacuum-assisted closure has been proven to help normalize the distribution of tension around the closed skin incision. This reduces the risk of wound dehiscence and scarring [4]. Until now, the use of NPWT was uncommon in breast surgery, especially in transgender top surgery, at least to our knowledge. As postoperative bleeding and revisional surgery, as well as wound dehiscence are common complications in transgender top surgery, we investigated NPWT as a kind of prophylactic wound dressing directly after top surgery in transgender patients.

## Methods

### Patient characteristics

In our retrospective study, we included all female-to-male (ftm) transgender patients, who underwent gender affirming breast surgery at the Medical University of Vienna, department of gynecology, between October 2017 and March 2020. Overall, 58 patients were examined, and gender-affirming hormone therapy was continuously administered.

The patients were operated mainly with two different techniques, periareolar (Picture 1) or common DIFNG scars (Picture 2); the surgeons decided between the techniques based on the breast size. All patients were aided with one drainage using vacuum on each side.

**Picture 1:**
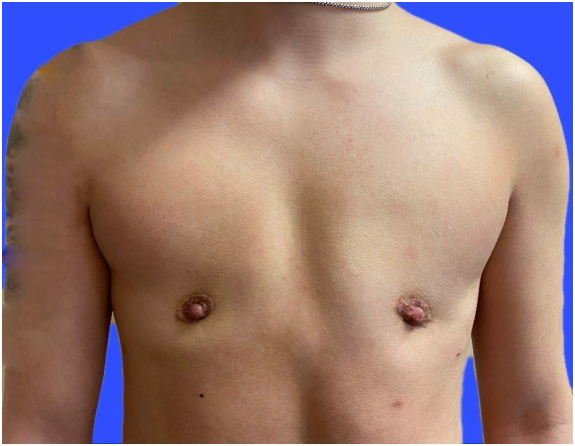
Periareolar Scar.

**Picture 2:**
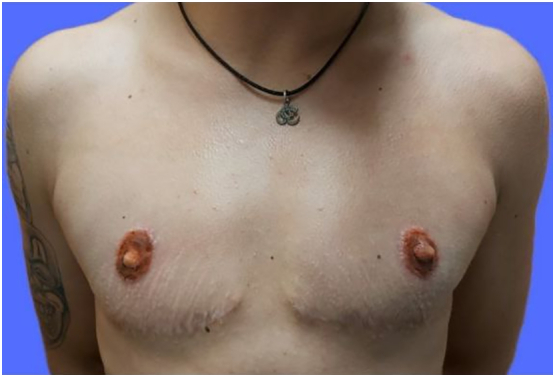
DIFNG scar.

The datas were collected 6 months after the operation. As complications we measured every intervention which needed an operation, stitches or emergency room visits.

### Dressing options

In the first one and a half years, the surgery was followed by a conventional dressing, which included adhesive strips, cotton and bandages for compression. In 2019, we mostly changed our postoperative dressing to NPWT. At our department, the common NPWT was with portable PICO Vac from Smith and Nephew. It is a portable NPWT system without an exudate can. The patients were treated with NPWT for 7 days including surgery day at 80 mmHg.

### Statistical analysis

Our main hypothesis was that NPWT is able to reduce complicationrates after gender affirming top surgery.

We tested the differences in relative frequencies of each type of complication – bleeding, seroma, and wound dehiscence – between conventional bandage or NPWT using either a Chi-squared test or an exact Fisher test, depending on whether any of the expected frequencies were < 5 or not. We tested the dependency of these differences on other variables, such as smoking status or surgical technique, using multi-variable generalized linear models. All statistical models were calculated using IBM SPSS, version 27.

## Results

Our 58 patients were between 17 and 50 years of age (mean: 25.7 years) and had a BMI between 15.94 and 36.33 kg/m^2^ (mean: 24.09 kg/m^2^).They were under androgenic hormonal therapy between 7 and 47 months (mean: 18.6) before surgery. Of the 58 patients, 15 required an DIFNG, while 43 had surgery using the periareolar technique. Postoperative complications included revision surgery due to bleeding, puncture due to seroma, and wound dehiscence. NPWT was applied in 23 patients (39.7 %), conventional dressing in 35 patients (60.3 %). Regarding the patient characteristics, it can be said that the patients in the group with NPWT had significantly lower BMI than those in the conventional group. Otherwise, there were no significant differences between the two dressing methods.

Picture 3 presents the main differences concerning the outcome complications. Of the 35 patients, 16 (45.7 %54.3 %) in the “conventional dressing” group experienced at least one complication, which was the case for 9 of 23 (39.1 %) in the “NPWT” group. This difference was not statistically significant. Regarding the type of complication, significant differences could be shown for revisions (2.9 % for conventional dressing vs. 30.4 % for pressure therapy, *p* = 0.005) and dehiscence (34.3 % for conventional dressing vs. 8.7 % for pressure therapy, *p* = 0.026), while the need for drainage procedures (20.0 % vs. 4.3 %, *p* = 0.129) was not statistically different.

**Picture 3:**
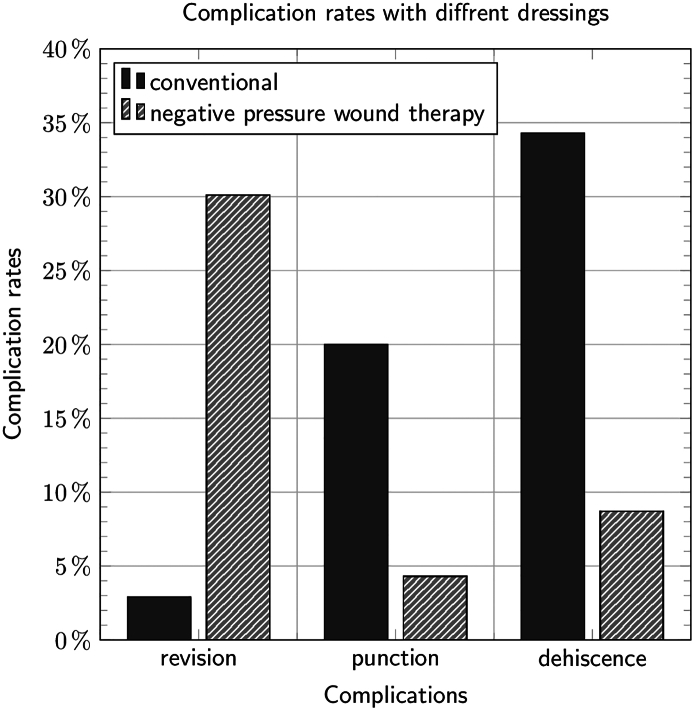
Differences concerning the outcome complications.

In 6.7 % patients in the periareolar group got a revision surgery compared to 16.3 % in the DIFNG group. There was also no difference of percutaneous drainage (13.3 % periareolar scar, 14.0 % DIFNG scar), but there is a tendence in dehiscences (6.7 % periareolar scar, 30.23 % DIFNG scar).

With regard to the two types of operation, periorarolar and DIFNG, there is no significant difference in the development of dehiscence and haematoma. The *p*-value 0.087 is to be seen as a trend, whereas in the one DFING group, consisting of one patient, dehiscences occur less frequently.

Since we found differences regarding smoking status and comorbidities showed differences between the two groups (see additional material), they were subsequently considered as potential confounders of the observed differences in outcome in a multi-variable generalized linear model. However, none of these variables significantly influenced the outcome, leaving the main observed differences with the type of dressing.

The probability of occurrence of dehiscence was calculated using regression analysis for a dichotomous outcome with the Firth log. Regression. Even when controlling for surgery type, it is significant that fewer dehiscences occur with NPWT (*p* = 0.046).

The likelihood of dehiscence occurrence was analyzed using regression analysis for a dichotomous outcome. The findings confirm that NPWT is associated with fewer cases of dehiscence, even when adjusting for surgical BMI. This effect remains statistically significant after controlling for BMI (*p* = 0.038).

## Discussion

Gender-affirming top surgery is on the rise [6]; hence, the search for the surgical method with the least complications. Transgender top surgery is connected with complications such as seroma, minor dehiscence, infection, and delayed healing [7].

Kim demonstrated that the use of incisional NPWT after expander-based breast reconstruction may be beneficial because it decreases the overall number of complications in comparison with standard wound treatment (*p* = 0.019), and skin necrosis is statistically significantly less frequent (*p* = 0.038) [8]. This was the same in our study, but with a one third higher rate of reoperations because of bleeding in the NPWT group. This effect of NPWT has never been described before. Perhaps higher rate of haematoma could be avoided when using an NPWT with less pressure or dressing the wounds the day after surgery. Another influencing factor regarding bleeding complications could be transgender hormonal treatment. Unfortunately, to the best of our knowledge there is insufficient data about this issue.

### Limitations

Our study has some limitations due to the small sample size and the retrospective design. The lack of a significant difference may be due to the fact that the study may not have had sufficient statistical power to detect a difference. We intend to conduct further prospective studies with more patients to determine whether NPWT can improve postoperative cosmetic outcomes and reduce the complication rate. The significantly lower BMI in the NPWT group represents a potential confounder, as a lower BMI could be associated with better wound healing. Adjustemnt of BMI could help to further investigate the effect.

## Conclusion

While on one hand we observed a reduction in wound healing problems using NPWT, unplanned return to the operating room because of severe hematoma was higher in this group.

## CRediT authorship contribution statement

**Carmen Leser:** Writing – original draft, Conceptualization. **Georg Dorffner:** Formal analysis. **Fiona Kabashi:** Writing – review & editing. **Christine Deutschmann:** Writing – review & editing, Data curation. **Daniel König:** Writing – review & editing. **Zaza Kashibadze:** Data curation. **Selina Ebner:** Writing – review & editing. **Daphne Gschwantler-Kaulich:** Writing – review & editing, Conceptualization.

## Ethics

All procedures performed in studies involving human participants were in accordance with the ethical standards of the institutional and/or national research committee and with the 1964 Helsinki declaration and its later amendments or comparable ethical standards. For this type of study formal consent is not required. It was approved by the local ethics committee (1548/2019).

## Funding

None.

## Declaration of competing interest

All authors declare that there are no conflict of interests regarding the paper above.
